# Medicine

**Published:** 1891-08

**Authors:** Wm. C. Krauss


### Progress in Medical Science.

### MEDICINE.

BY

WM. C. KRAUSS, M. D.

In an able article on Crime and Responsibility, by Daniel Clark, M. D., in the April number of the *American Journal of Insanity*, the following deductions are obtained :

1. The natural history of crime shows that brains of chronic criminals deviate from the normal type and approach those of the lower creation.

2. That many such are as impotent to restrain themselves from crime as the insane.

3. That crime is an ethical subject of study outside of its penal relations. That insanity and responsibility may exist. That some insane can make competent wills, because rational. Those classes of humanity exempt from responsibility are: first, the child of immature age; second, the idiot; third, the imbecile; fourth, various classes of the insane; fifth, the epileptic, when fits are coming on or leaving; sixth, the dipsomaniacal in the frenzy of drunkenness.

HESSELIEVITCH — *Ibid* — studied the influence of stomach lavage upon the assimilation of fats in five healthy individuals. Each experiment lasted eighteen days, and was divided into three periods of six days each. The lavage occurred half an hour before dinner, the water being 28° to 30° centigrade. In four cases the author noted an increase in the assimilation of fatty matter, and in one case a decreased assimilation. This effect continued for some time after the lavage.

---

IN *The American Meteorological Journal* for May, 1891, is an interesting article by Hildebrandsson, on "Is the Influenza Spread by the Wind?" He takes up the course of the epidemic, beginning in St. Petersburg on the 15–17 of November, 1890, then follows it to Hamburg, where it first appeared December 1st, and Paris December 15th. All reports from the newspapers agreed that the influenza spread from east to west, in which case the infection had gone *against* the wind, since at this time there was not a single easterly wind over the countries about the Baltic sea. It appeared in Stockholm on the 27th of November, and was, therefore, impossible that the infection could have been imported from Russia by the wind.

The result of this research is, of course, that *the influenza is propagated by infection, and conducted from place to place through human circulation, and that the time of incubation is two to three days.*

The state of the weather seemed to have had no influence on this sickness. In fact, the influenza raged with the same severity in countries possessing very different climates, and during very different weather conditions.

It seemed unnecessary for a person to be in direct contact with a sick person for infection. On the contrary, it is almost certain that the infecting germs can be transported with corpses, clothes, etc. A curious fact has been related to me by Professor J. Hann, of Vienna. At Christmas, when Vienna was scourged by the influenza, two gentlemen went up to the meteorological observatory at the top of the Sonnblick (10,000 feet). The single observer living at this height, isolated from the world, fell sick of influenza a few days after their visit. Of course the visiting gentlemen were in the best of health, since they could ascend a mountain of 10,000 feet in winter.

